# Production and characterization of *in planta* transiently produced polygalacturanase from *Aspergillus niger* and its fusions with hydrophobin or ELP tags

**DOI:** 10.1186/1472-6750-14-59

**Published:** 2014-06-27

**Authors:** Eridan Orlando Pereira, Igor Kolotilin, Andrew Jonathan Conley, Rima Menassa

**Affiliations:** 1 Agriculture and Agri-Food Canada, 1391 Sandford Street, London, ON N5V 4T3, Canada; 2 Department of Biology, Western University, London, ON N6A 5B7, Canada; 3 VTT Technical Research Centre of Finland, Tietotie 2, Espoo 02044, Finland; 4 Current address: Universidade Estadual do Ceará, Av. Dr. Silas Munguba, 1700 - Campus do Itaperi, Fortaleza 60714-903, Brazil

**Keywords:** Polygalacturonase, *Aspergillus niger*, *Nicotiana benthamiana*, Elastin-like polypeptide, Hydrophobin I, Cellulosic biofuels

## Abstract

**Background:**

Pectinases play an important role in plant cell wall deconstruction and have potential in diverse industries such as food, wine, animal feed, textile, paper, fuel, and others. The demand for such enzymes is increasing exponentially, as are the efforts to improve their production and to implement their use in several industrial processes. The goal of this study was to examine the potential of producing polygalacturonase I from *Aspergillus niger* in plants and to investigate the effects of subcellular compartmentalization and protein fusions on its accumulation and activity.

**Results:**

Polygalacturonase I from *Aspergillus niger* (*An*PGI) was transiently produced in *Nicotiana benthamiana* by targeting it to five different cellular compartments: apoplast, endoplasmic reticulum (ER), vacuole, chloroplast and cytosol. Accumulation levels of 2.5%, 3.0%, and 1.9% of total soluble protein (TSP) were observed in the apoplast, ER, and vacuole, respectively, and specific activity was significantly higher in vacuole-targeted *An*PGI compared to the same enzyme targeted to the ER or apoplast. No accumulation was found for *An*PGI when targeted to the chloroplast or cytosol. Analysis of *An*PGI fused with elastin-like polypeptide (ELP) revealed a significant increase in the protein accumulation level, especially when targeted to the vacuole where the protein doubles its accumulation to 3.6% of TSP, while the hydrophobin (HFBI) fusion impaired *An*PGI accumulation and both tags impaired activity, albeit to different extents. The recombinant protein showed activity against polygalacturonic acid with optimum conditions at pH 5.0 and temperature from 30 to 50°C, depending on its fusion. *In vivo* analysis of reducing sugar content revealed a higher release of reducing sugars in plant tissue expressing recombinant *An*PGI compared to wild type *N. benthamiana* leaves.

**Conclusion:**

Our results demonstrate that subcellular compartmentalization of enzymes has an impact on both the target protein accumulation and its activity, especially in the case of proteins that undergo post-translational modifications, and should be taken into consideration when protein production strategies are designed. Using plants to produce heterologous enzymes for the degradation of a key component of the plant cell wall could reduce the cost of biomass pretreatment for the production of cellulosic biofuels.

## Background

The rising demand for sustainable energy in the world and the availability of abundant biomass have attracted an increasing interest in bioconversion of plant cell walls into ethanol and other biofuels [1]. There are many challenges to developing the capability for producing ethanol in an effective and economical fashion. After years of research on the production of ethanol from lignocellulosic biomass, one of the key impediments in this field is the recalcitrance of the plant cell wall to breakdown. The digestibility of cellulose present in lignocellulosic biomass is hindered by many physicochemical, structural, and compositional factors [2] and current pretreatments rely on thermochemical technologies using high temperatures, toxic acids, peroxides, and ammonia. The goal of pretreatment is to break down the physical structure of the cell wall and make the crystalline structure of cellulose more accessible to cellulases. This pretreatment process may account for up to 30% of the cost of biofuel production [1]. Enzymatic pretreatment represents a more promising and environmentally friendly technology.

The plant cell wall is mainly composed of cellulose, hemicellulose, lignin, pectin and smaller amounts of several other inorganic materials [3]. A cell wall component that, particularly in dicots, is critical for tissue integrity and accessibility to cell wall-degrading enzymes (CWDEs) is the cohesive pectin matrix embedding the cellulose-hemicellulose network that confers rigidity to the cell wall [4]. Pectin is the most complex class of plant cell wall polysaccharides comprised mainly of three pectic polysaccharides named homogalacturonan (HG), rhamnogalacturonan I and rhamnogalacturonan II [5]. Complete pectin degradation requires a battery of pectinases, including pectate lyases, polygalacturonases (PGs) and rhamogalacturonases, pectin methylesterases and pectin acetylesterases. However polygalacturonases, the enzymes responsible for catalyzing hydrolysis of α-1,4-glycosidic linkage in the α-(1,4)-linked D-galactopyranosyluronic acid (Gal*p* A) residues of HG, are considered a key factor in plant tissue maceration, especially during phytopathogen infection [6]. It has been observed that the addition of a pectinase cocktail, which has PG as a component, to cellulase cocktails increases the yields of glucose by 7.5% [7]. However, due to the different degree of acetylation of the Gal*p* A units in the HG region it is essential that the candidate enzyme presents a certain degree of flexibility for efficient hydrolysis. *In silico* models in agreement with mass spectrometry studies using 3 different PGs demonstrated that PG I from *Aspergillus niger* (*An*PGI) has a higher tolerance towards acetylated pectin [8].

The cost of biomass degrading enzymes is widely considered an important factor in the commercialization of lignocellulosic biomass-to-ethanol processes and the use of microorganisms in the production of enzymes is the main platform currently available. One of the reasons for high enzyme cost is the need for high volume bioreactors which substantially increase the cost of production. The use of plants represents an alternative for enzyme production [9]. Transient expression of recombinant proteins in *Nicotiana benthamiana* constitutes an ideal system for screening and analysis of recombinant enzymes due to its low cost and short time of production, where from cloning to expression data can be achieved in only two weeks. The most promising enzymes and constructs are then usually stably transformed into plants, which offer an almost unlimited scale-up potential. Nonetheless, depending on the purpose, large scale protein production using transient expression systems has been reported [10], where 450–750 kg of *N. benthamiana* biomass can be infiltrated in 8 hours, producing 1–10 g of recombinant protein/kg of fresh weight within 7–14 days [11].

Although the expression of CWDEs in plants has been demonstrated, accumulation levels in nuclear transformed plants were low [12] and due to the complexity of the plant cell wall, other enzymes remain to be explored using this system. Several strategies have been proposed to improve protein production in plants. Among them, protein fusions can address issues of stability and aid in purification [13]. Targeting heterologous proteins to the appropriate subcellular compartment can be critical for obtaining high levels of accumulation, since the structure and stability of the recombinant protein is affected by its route and final destination in the cell. In plant leaves, heterologous proteins have been typically targeted to the apoplast, ER, vacuole, chloroplasts and cytosol [14] by including a combination of targeting and retention signal sequences in the expression construct.

In the case of protein fusions, elastin-like polypeptide (ELP), a pentapeptide repeat polymer (Val-Pro-Gly-Xaa-Gly) that forms an aggregate above its transition temperature and hydrophobin (HFBI), a small and surface-active protein derived from filamentous fungi have proven valuable for improving recombinant protein accumulation in plants. Despite the fact that these peptides were originally designed for purification, they have also been shown to increase recombinant protein accumulation in plants [15-17].

Using a series of constructs targeting the polygalacturonase I from *Aspergillus niger* (*An*PGI) to different subcellular compartments, we report here the effects of subcellular targeting on the accumulation and activity of *An*PGI in leaves of *N. benthamiana*. We also analyze the effects of ELP and HBFI on protein accumulation in the ER and vacuole. Lastly we show through self-hydrolysis analysis that leaves producing *An*PGI have a higher content of reducing sugars (up to 20 fold more) in comparison with wild type leaves. These results provide an important step towards inexpensive production of cell wall degrading enzymes for the bioconversion of biomass into fermentable sugars.

## Results

### Analysis of heterologous polygalacturonase I transiently expressed in *N. benthamiana*

**Figure 1:**
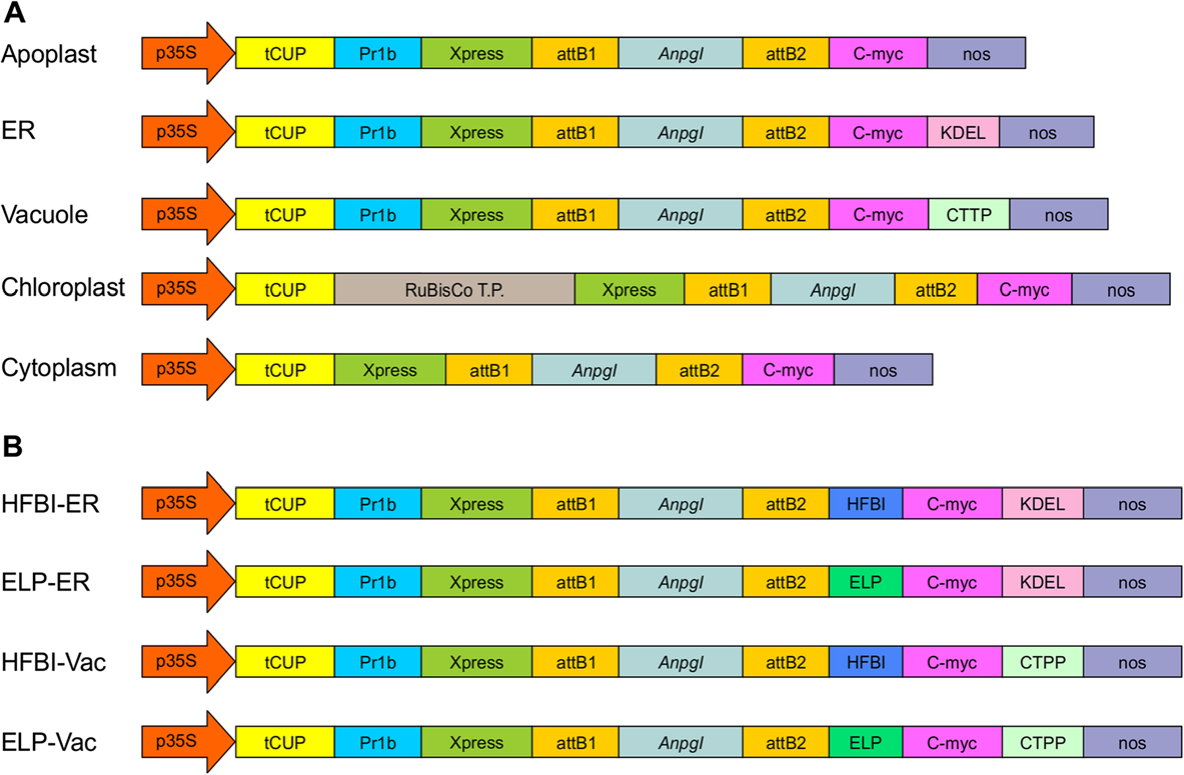
**Schematic representation of the diverse *An*PGI heterologous expression constructs evaluated in this study: A) constructs used for protein targeting experiments and B) constructs used to analyze the effect of fusion tags on protein expression.** p35S, double enhanced 35S promoter from Cauliflower Mosaic Virus 35S gene; tCUP, translation enhancer from the tobacco cryptic upstream promoter; nos, nopaline synthase transcription terminator; Pr1b, tobacco pathogenesis related 1b protein secretory signal peptide; C-myc, detection/purification tag; KDEL, endoplasmic reticulum retrieval tetrapeptide; CTPP, vacuole sorting peptide; RuBisCo T.P., rubisco small subunit transit peptide; HFBI, hydrophobin I; ELP, elastin-like polypeptide. Schematic not drawn to scale.

Several factors need to be taken into consideration for achieving high levels of recombinant protein accumulation in plants, including high transcript levels, correct post translational modifications, and protein turnover. To maintain high levels of transcripts, the double enhanced cauliflower mosaic virus (CaMV) 35S constitutive promoter was used in a series of *AnpgI* gene expression vectors that were designed to target the recombinant protein to five different subcellular compartments: apoplast, ER, vacuole, chloroplasts and cytosol (Figure 1A). Agrobacterium cultures containing each of these vectors were transiently co-infiltrated in *N. benthamiana* with an Agrobacterium culture containing a p19-encoding construct. P19 is a suppressor of post-transcriptional gene silencing from *Cymbidium* ringspot virus [18]. Leaves infiltrated only with p19 were used as the negative control.

Overexpression of recombinant proteins tends to result sometimes in chlorosis or even necrosis of the infiltrated leaf sectors [15]. Although pectic enzymes targeted to the apoplast in transgenic plants have been shown to impair plant growth and fitness [19], leaves infiltrated with the *An*PGI constructs did not show any chlorotic or necrotic phenotype by harvest time.

Analysis of transiently expressed protein in *N. benthamiana* was performed by western blot analysis of leaf total soluble protein extracts, using a monoclonal antibody against the c-Myc tag to determine *An*PGI accumulation.

**Figure 2:**
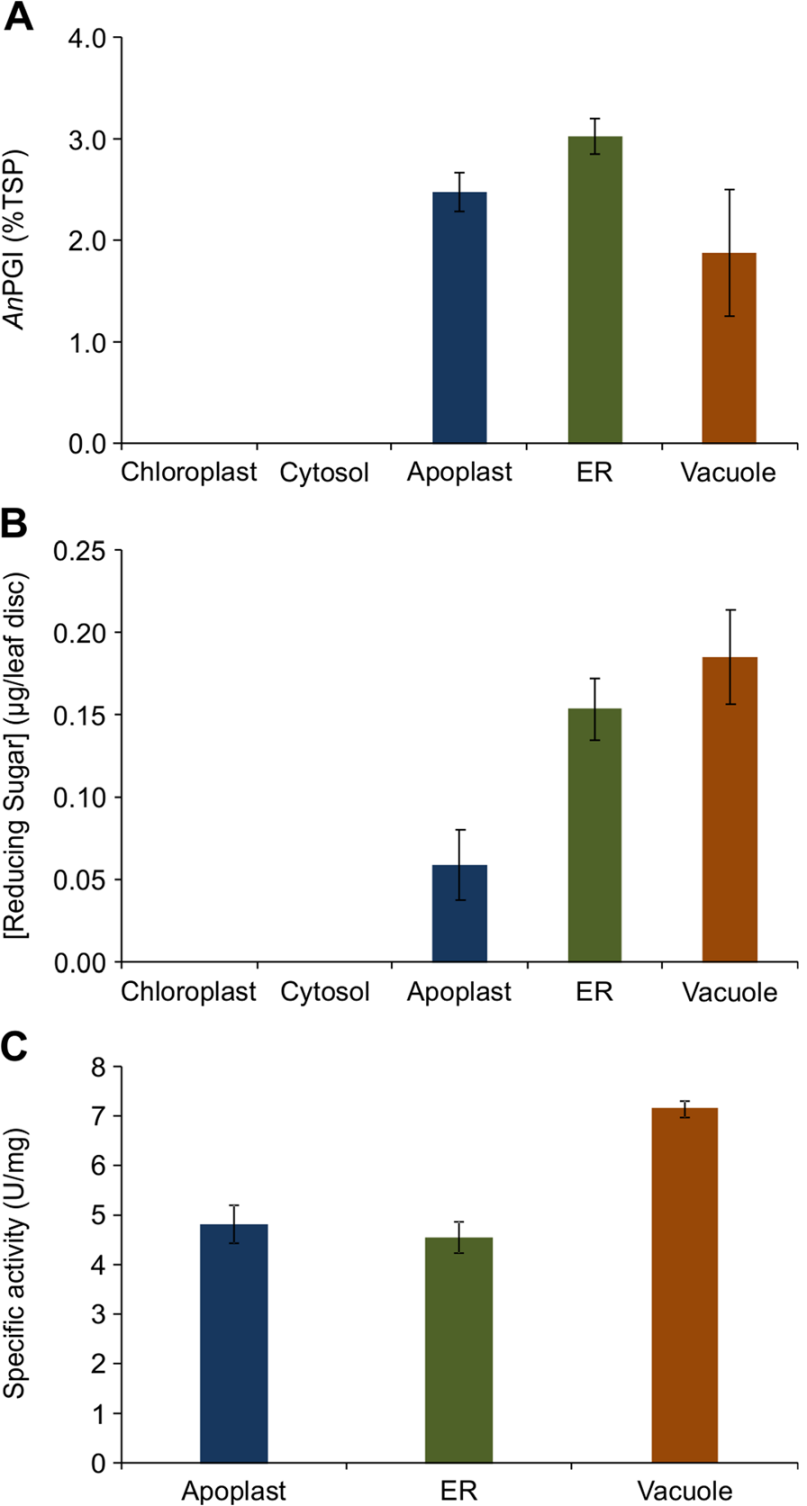
**Accumulation and activity of *An*PGI in different subcellular compartiments. A)** Accumulation of *An*PGI in different subcellular compartments. **B)** A *N. benthamiana* agroinfiltrated leaf disc was incubated in 50 mM sodium acetate at 50°C for 24 h and used to analyze self hydrolysis and release of reducing sugars using the dinitro salicylic acid (DNS) method. **C)** Purified *An*PGI was used to confirm the difference in polygalacturonase activities when targeted to different subcellular compartments using polygalacturonic acid as the substrate. Accumulation results represent the average of *An*PGI levels in five different plants, and the release of reducing sugars and protein activity were determined in triplicate. Reducing sugar concentration was normalized using the assay results for wild-type plants. Error bars represent ± standard error.

Densitometry analysis showed high accumulation of *An*PGI at 2.5%, 3.0% and 1.9% of total soluble protein (TSP) in the apoplast, ER and vacuole respectively (Figure 2A). These analyses also demonstrated that the *An*PGI recombinant protein does not accumulate to detectable levels when targeted to the chloroplasts or cytosol of *N. benthamiana* leaves. Accumulation of *An*PGI only occurred in the secretory pathway, and the fact that *An*PGI has two *N*-glycosylation sites led us to ask if glycosylation may be related to stability of the protein.

### Recombinant *An*PGI glycosylation analysis

**Figure 3:**
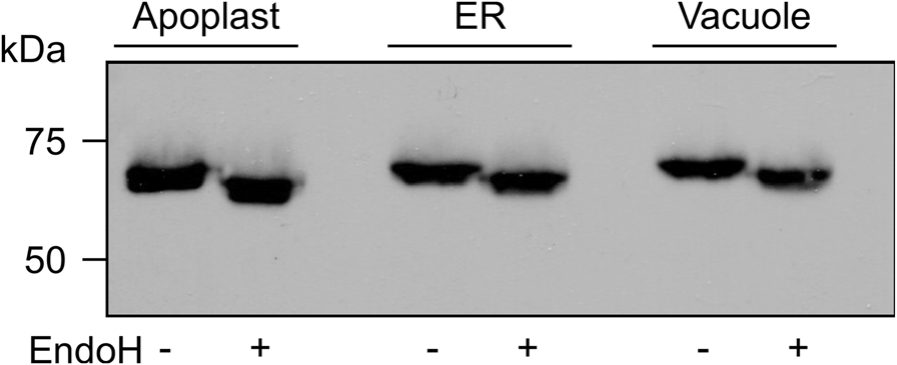
**Deglycosylation analysis of plant-expressed *An*PGI targeted to different compartments.** Purified *An*PGI was incubated for 3 h in denaturing conditions in the absence (-) or presence (+) of EndoH, analysed by SDS-PAGE under reducing conditions, and subjected to Western blot analysis with anti-C-myc antibody.

To determine the presence of glycans, a deglycosylation experiment on purified proteins was performed by digesting *An*PGI with Endoglycosidase H (Endo H) followed by SDS-PAGE and western blot analysis. After deglycosylation with Endo H, the downshift in the band size indicates that *N*-glycans were incorporated in the protein (Figure 3). Since *N*-glycosylation does not occur in the cytosol or chloroplast, this data suggests that glycosylation could be essential for the *in vivo* stability of *An*PGI. A similar effect was observed for the expression of *Acidothermus cellulolyticus* E1 endo-*β*-1,4-glucanase in tobacco leaves [20]. Although in that case, the protein was observed in the cytosol and chloroplasts but with average levels of 0.004 and 0.0003% of TSP respectively, which was more than 100 fold lower than its accumulation in the secretory pathway.

Results of western blot analysis showed that plant-produced *An*PGI has a molecular mass of about 60 kDa (Figure 3). This molecular mass is substantially larger than the predicted molecular weight of *An*PGI constructs of 41.9 kDa (36.4 kDa of *An*PGI plus 5.5 kDa of the added tags). This result is in accordance with previous results reported in the literature, where a 55 kDa molecular mass was determined for *An*PGI by SDS-PAGE [21]. This difference may reflect the acidic nature of *An*PGI protein, which has an estimated pI of 4.0.

### The influence of subcellular targeting on *An*PGI activity

*N. benthamiana* leaves producing *An*PGI were analyzed for self-hydrolysis by incubating leaf discs at 50°C for 24 h and using the dinitrosalicylic acid (DNS) assay. As expected, no significant difference in the release of reducing sugars was observed in leaves infiltrated with constructs targeting the chloroplasts or cytosol, compared with the negative control infiltrated with p19 alone. However, reducing sugars were released from tissues accumulating *An*PGI in the apoplast, ER and vacuole. Also, a higher amount of reducing sugars was released when *An*PGI was targeted to the vacuole, despite the lower amount of accumulated protein compared with the apoplast and ER (Figure 2A and B). This result could mean that post-translational modifications specific to the vacuole might affect the activity of the enzyme, or that the acidic environment in the vacuole is more conducive to proper folding of *An*PGI, and led to a more active enzyme. This led us to determine the specific activity of purified *An*PGI targeted to the three compartments.

Transiently produced *An*PGI was purified by affinity chromatography using the c-Myc tag fused at the C-terminus of the protein, and purified protein was used for a comparative analysis of *An*PGI catalytic activity when localized to the apoplast, ER or vacuole. The results showed that indeed the specific activity of *An*PGI against polygalacturonic acid is 30% higher when the protein is targeted to the vacuole (Figure 2C) than when it is targeted to the apoplast or ER.

### The effect of ELP and HFBI fusions on *An*PGI accumulation

Elastin-like polypeptides (ELPs) are pentapeptide repeat polymers of Val-Pro-Gly-Xaa-Gly, where the guest residue Xaa can be any amino acid except proline [22], that aggregate above a transition temperature, T_t_. Due to this property, ELPs have been explored as fusion partners for an inexpensive non-chromatographic method for protein purification [16]. Besides their utility for purification, ELP fusions have been shown to increase accumulation levels of several heterologous proteins by 2- to 100-fold when expressed in plants [16,17].

Hydrophobins are small fungal proteins with a size of approximately 10 kDa, which contain a large proportion of hydrophobic residues and eight cysteines connected by disulfide bonds [23]. Due to their propensity to self-assemble into an amphipathic protein membrane at hydrophilic-hydrophobic interfaces, and their ability to alter the hydrophobicity of their fusion partners, hydrophobins have been explored for purification purposes [23]. Hydrophobins were also demonstrated to be useful in improving the accumulation of fusion partners [24]. For example, a hydrophobin I (HFBI) gene from *Trichoderma reesei* was used as a green fluorescent protein (GFP) fusion which led to an increase of GFP accumulation in *N. benthamiana* leaves from 18% to 38% of TSP [15].

**Figure 4:**
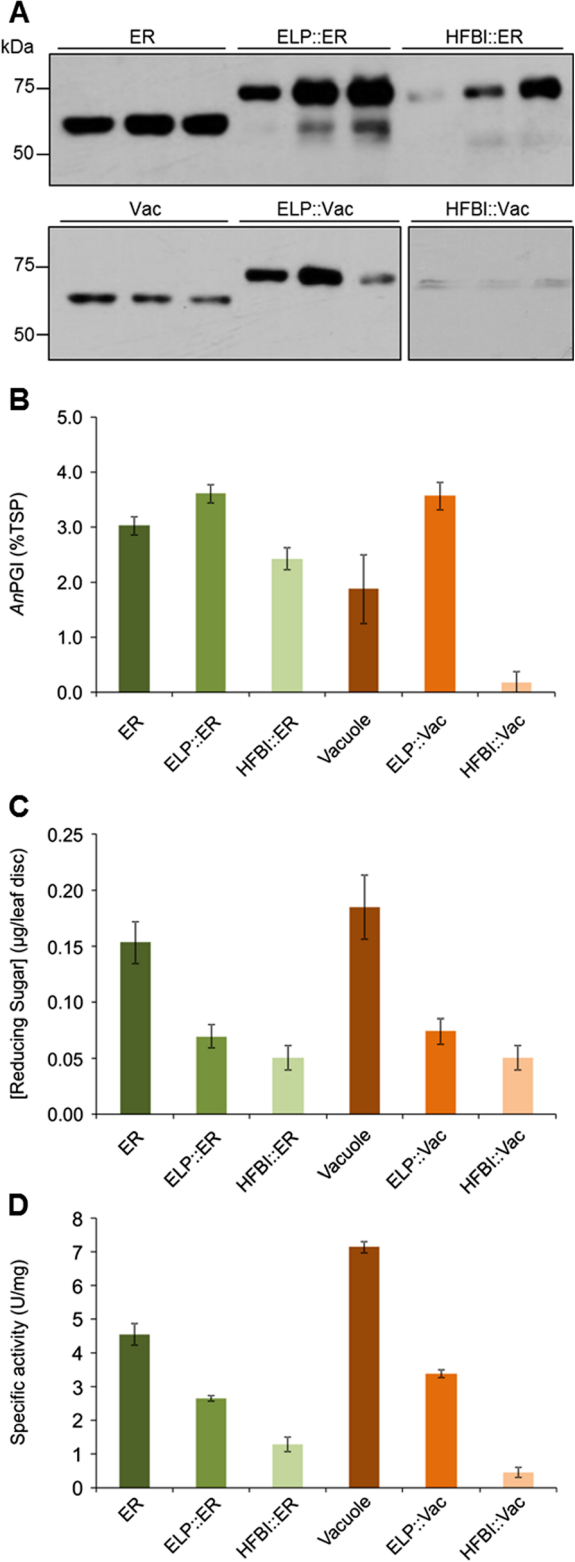
**Accumulation and activity of *An*PGI and its fusions with ELP and HFBI targeted to the ER and vacuole. A)** Western blot analysis of AnPGI and its fusions targeted to the ER or the vacuole. Each lane was loaded with 5 μg TSP from three replicates, and detected with anti c-myc antibody. **B)** The effect of fusion partner on the accumulation of *An*PGI. **C)** A *N. benthamiana* leaf disc agroinfiltrated with *An*PGI and its fusions was incubated in 50 mM of sodium acetate at 50°C for 24 h and used to analyze self hydrolysis and release of reducing sugars using the dinitro salicylic acid (DNS) method. **D)** The specific activity against polygalacturonic acid was used to analyse the effect of ELP and HFBI fusions on *An*PGI activity. Accumulation results represent the average of *An*PGI levels in five different plants, and the release of reducing sugars and protein activity were determined in triplicate. Reducing sugar concentration was normalized using the assay results for wild-type plants. Error bars represent ± standard error.

To analyze the effects of ELP and HFBI fusions on *An*PGI accumulation, fusion constructs (Figure 1B) were transiently co-expressed in *N. benthamiana* with p19. All proteins were produced, and although some degradation was observed with the ELP-ER and HFBI-ER constructs, most of the protein was in full size form (Figure 4A). Plants analyzed 4 days post infiltration showed a higher accumulation of *An*PGI fusion with ELP in both the ER and vacuole when compared with unfused *An*PGI (Figure 4B). Although the ELP fusion enhanced *An*PGI accumulation in both compartments, the effect of the fusion was more significant in the vacuole, where the ELP enhanced the accumulation of *An*PGI by almost 2-fold from 1.9 to 3.6% of TSP (Figure 4B). The presence of HFBI on the other hand was deleterious to the accumulation of *An*PGI in both compartments, with very low accumulation in the vacuole.

To further characterize the effects of the protein fusions, we assessed *in planta* self-hydrolysis and enzymatic activity for *An*PGI::ELP and *An*PGI::HFBI. We found that the presence of protein fusions impaired the activity of *An*PGI resulting in a reduction of the amount of reducing sugars released *in planta* (Figure 4C). The same reduction was confirmed with the purified enzyme against its substrate, polygalacturonic acid (Figure 4D).

### Biochemical characterization of recombinant *An*PGI and its fusions

**Figure 5:**
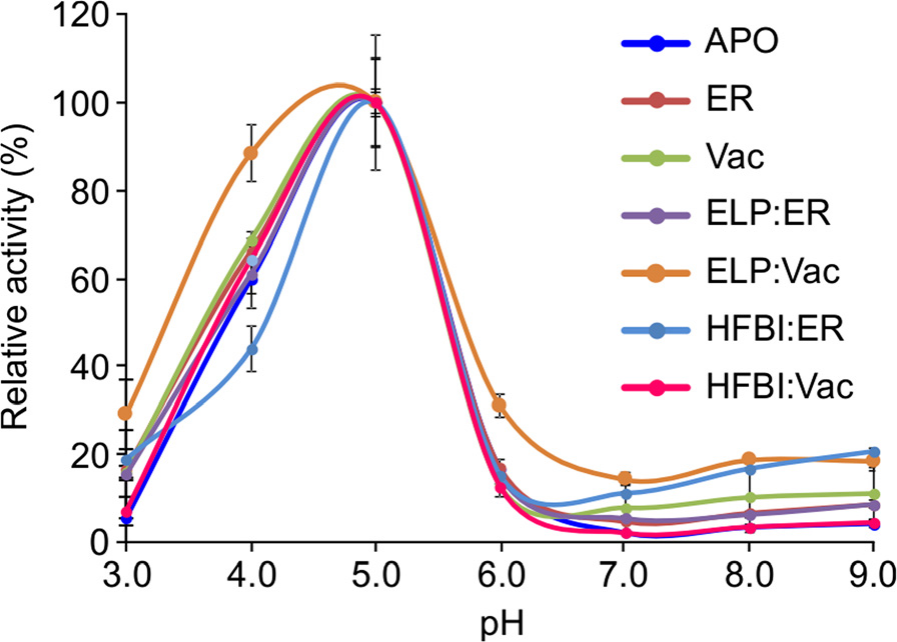
**The pH profile of *An*PGI transiently expressed in *N. benthamiana *leaves.** The purified polygalacturonase, *An*PGI and its fusions with ELP or HFBI were assayed at pH ranging from 3.0 to 6.0, in 50 mM sodium acetate buffer and at pH 6.0 to 9.0, in 50 mM sodium-phosphate buffers at 40°C. Each point was determined in triplicate and shown as the average ± standard error.

**Figure 6:**
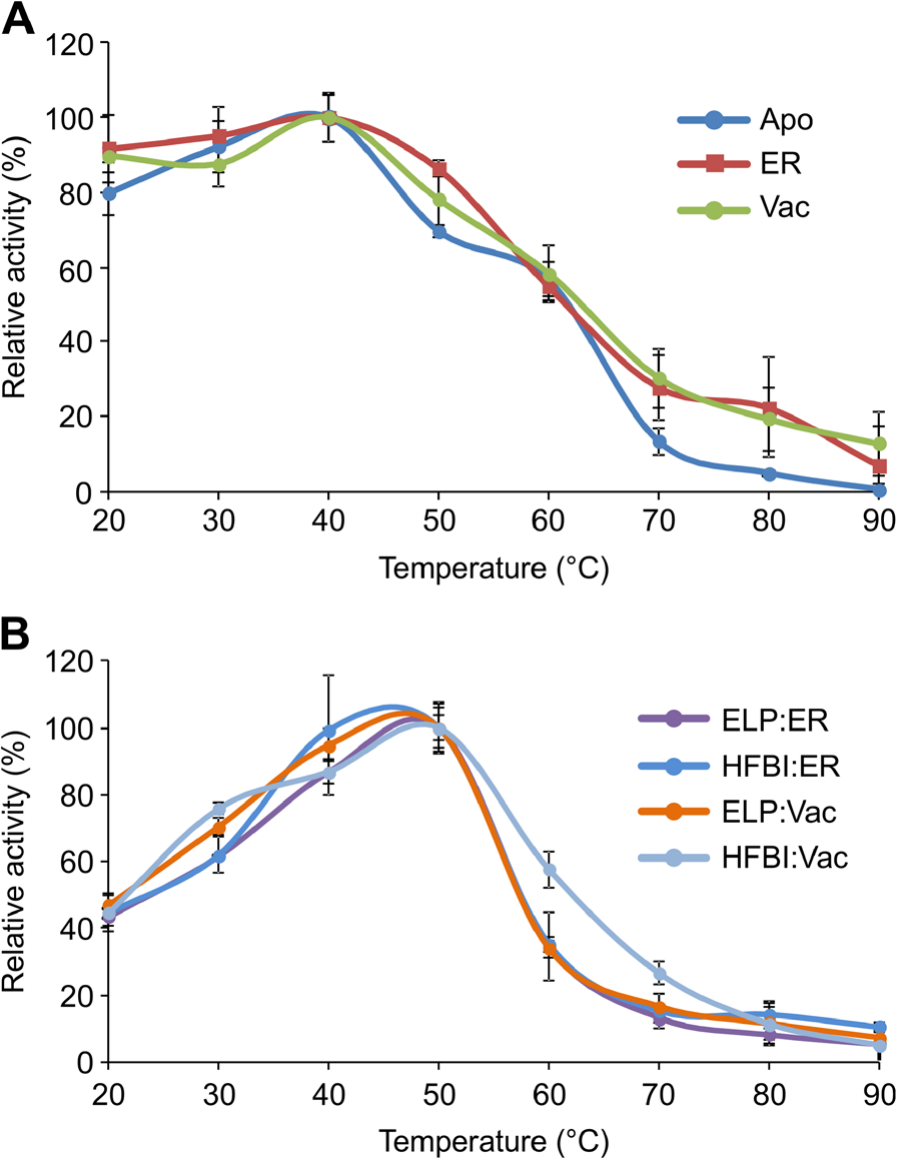
**Temperature profile of *An*PGI transiently expressed in *N. benthamiana *leaves. A)** Purified polygalacturonase I targeted to different subcellular compartments and **B)***An*PGI fusions with ELP or HFBI targeted to the ER and vacuole. The enzymes were assayed at pH 5.0 and determined as the release of reducing ends of the 0.5% polygalacturonan solution in 50 mM sodium acetate buffer. The highest polygalacturonase activity is set to 100%. Each point was determined in triplicate and shown as the average ± standard error.

Temperature and pH optima of purified plant-produced *An*PGI were determined and were compared with previously described results of *Aspergillus niger*-produced enzyme [25]. Recombinant *An*PGI, independent of its subcellular localization, showed similar biochemical properties as the native *Aspergillus* enzyme with respect to its pH optimum (pH 5.0) (Figure 5). However, the temperature optimum was found to be at least 10°C lower than results previously reported for native *An*PGI [25], presenting 80-100% activity at a broad temperature range, from 20-40°C (Figure 6A).

Temperature and pH optima of the ELP and HFBI fusion proteins were compared with those of unfused *An*PGI. No differences of activity at various pH values were observed, with all proteins showing a pH optimum at pH 5.0 (Figure 5). However, the fused *An*PGI recovered its native temperature optimum of 50°C and displayed a narrower temperature range than the unfused enzyme (Figure 6B).

## Discussion

Because of the increased interest in the conversion of biomass to biofuels, several enzymes useful in the bioconversion of plant tissues to biofuels have already been expressed using plants as bioreactors. However, the main emphasis until now has been on the expression of cellulases and xylanases targeting cellulose and hemicellulose [26]. Pectins, especially in dicots, compose 35% of primary plant cell walls and up to 5% of walls in wood tissue [27] and are critical for tissue integrity and accessibility to cell wall-degrading enzymes (CWDEs). Previous studies have focused on the heterologous expression of polygalacturonase as a bioregulator for pathogen defense or as a modifier of plant cell wall pectin structure [4,28]. To test the potential of plants as bioreactors for the production of polygalacturonase, we produced *Aspergillus niger* polygalacturonase I (*An*PGI) in *Nicotiana benthamiana* and characterized the produced enzyme.

The subcellular accumulation of *An*PGI was higher in the ER, followed by the apoplast and vacuole while no detectable *An*PGI was found when targeted to the chloroplasts or cytosol. These results confirm the impact of different subcellular compartments on the accumulation of cell wall degrading enzymes, especially in the case of a protein that requires post-translational modifications. Similar results have been previously described for another *Aspergillus niger* cell wall degrading enzyme, *β*-glucosidase I, produced in stably transformed tobacco plants [29]. *β*-glucosidase I accumulated to very low levels when targeted to the chloroplasts or cytosol, and was not functional possibly due to lack of glycosylation [29]. The glycosylation process is initiated during protein translocation into the ER and the addition of *N*-glycans to the protein has been linked to diverse roles, including stability against denaturation and proteolysis, fine-tuning the charge and isoelectric point, regulating protein turnover [30], and has also been linked to protein activity [31]. Although some glycoproteins may accumulate well in the cytosol [32] and chloroplasts [33], and perform the required biological functions, others such as *An*PGI may require glycosylation for stability and will not accumulate in those compartments.

The subcellular location of *An*PGI within the secretory pathway had a distinct effect on its specific activity. *An*PGI targeted to the vacuole showed 30% higher specific activity than *An*PGI targeted to the apoplast or ER. This result is consistent with at least two other CWDEs produced in sugar cane, where activities of cellobiohydrolase I (CBH I) and CBH II were, respectively, five and two times more active when targeted to the vacuole [34] when compared with ER-targeted proteins. As well, the effect of subcellular targeting on protein activity has been observed with human gastric lipase produced in tobacco; however, in this case, the vacuolar-targeted enzyme exhibited two-fold lower specific activity compared with the same protein targeted to the apoplast or ER, probably due to proteolytic-induced instability of this specific protein when targeted to the vacuole [35]. In the case of glycosyl hydrolases, higher specific activity was expected when targeted to the vacuole, as proteomic analysis of the vegetative vacuole of *Arabidopsis thaliana* uncovered several glycosyl hydrolases, including a polygalacturonase [36], which indicates that the vacuole is a natural compartment for these enzymes and may provide a chemical environment that favors appropriate protein folding and activity. Therefore, and although most of the attention when analyzing protein expression in plants has been directed towards protein accumulation, protein activity may play a bigger role in the final analysis, especially when compartmentalization results in protein with low or no activity, which in many cases is due to a lack of post-translational modifications or protein truncation [29]. When compared to the previously reported specific activity of native *An*PGI [21], plant-produced *An*PGI showed 75 times lower specific activity. However, direct comparison of specific activities of enzymes characterized in different studies is problematic due to experimental differences as was pointed out by Kester et al. [21] and a side-by-side comparison would need to be conducted.

Recently, diverse protein fusions have been explored to increase the level of heterologous protein production in plants using technologies such as Zera [37], ELPs [16,38] and HBFI [15]. The advantage of using these protein fusions is that besides enhancing protein accumulation in plants, they also provide a means for their purification. In the study presented here, ELP and HFBI tags were fused to *An*PGI and targeted to the ER and vacuole. *An*PGI::ELP transient expression in *Nicotiana benthamiana* leaves led to an increase in accumulation in both compartments, while *An*PGI::HFBI transient expression led to a small reduction in the ER and to a drastic reduction in the vacuole. This is the first report of ELP and HFBI fusions targeted to the vacuole and the observation that ELP increases accumulation while HFBI reduces accumulation is interesting and should be further investigated. One of the hypotheses for the lower accumulation of *An*PGI::HFBI is that HFBI, which is a globular protein with four disulfide bonds [39], might be affecting *An*PGI folding, leading the misfolded protein to ER-associated protein degradation (ERAD). Specifically for this study, a flexible linker consisting of (GGGS)_3_ was used to separate HFBI from *An*PGI and was probably not effective enough in separating the two protein domains. One of the possible solutions for this problem is the optimization of the linker used to separate the two proteins. Optimization of linkers has been shown to improve both protein expression yield and biological activity [40]. A recent study comparing several linkers demonstrated the need for empirical evaluation of different linkers, and the possible beneficial effect of twistable linkers on activity of the fusion partners, possibly by allowing a reorientation of the functional domains [32]. Moreover, the addition of ELP and HFBI fusions had adverse effects on enzyme activity. Although these results were not predicted, they were not completely unexpected. Previous studies using ELP tags have shown different outcomes on protein activity [38,41] and impact of fusion tags on recombinant protein activity needs to be assessed on a case-by-case basis.

More specifically in the case of *An*PGI::ELP targeted to the vacuole, because the loss of activity was directly proportional to the increase in accumulation, the ELP fusion may still offer a viable option in the production of this enzyme for industrial purposes. ELPs are thermally responsive synthetic biopolymers [22] that are valuable for simple nonchromatographic bioseparation of recombinant proteins. Because of this property, and taking into consideration the amount of enzyme that will be necessary for the production of soluble sugars, the use of this technology could represent a potential solution to lower the enzyme cost in the process by recycling the enzyme of interest and should be further investigated.

As the HFBI fusion can also be used for bioseparation [24], the same mechanism could be used for recycling the enzyme; however in this case, as HFBI showed a detrimental effect on both protein accumulation and activity of *An*PGI, improvements to the construct design for increasing accumulation of the protein fusion should be assessed prior to further developing this technology.

Although in the long term, *An*PGI should be produced directly into energy crops to reduce the costs of production of second generation biofuels, the results achieved here represent a step forward towards the development of this technology. The selection of the best compartment to store and maintain bioactive enzymes was demonstrated, no necrosis was observed in plants producing *An*PGI, indicating that this enzyme was not toxic at the accumulation levels obtained and can be potentially produced in stable transgenic plants. However, as the expression of pectic enzymes in the apoplast has been shown to affect plant growth and fitness [19,42] , genetic engineering strategies like the use of inducible promoters should be explored in order to avoid negative effects on plant growth. Further, storing this enzyme in the ER or vacuole of stably transformed plants could prevent negative effects on growth and should be explored. Moreover, the combination of plant-produced cell wall degrading enzymes with an ELP fusion can be promising for reducing the costs associated with enzymatic deconstruction of plant cell walls.

## Conclusions

In conclusion, this study demonstrated the feasibility of using plants as bioreactors for the production of active *An*PGI at high levels. Both the accumulation and bioactivity of plant-produced *An*PGI were affected by different sub-cellular targeting of the recombinant protein, as well as by fusion with ELP and HFBI. Such analyses allow us to select the best combination of a sub-cellular compartment and a fusion partner for production of enzymes in plants, thus maximizing their catalytic potential in downstream applications.

## Methods

### *AnpgI* plant expression vectors

The polygalacturanase I gene from *A. niger* (Accession # XP_001389562) was chemically synthesized (GenScript, Piscateway, NJ, USA) (*AnpgI*) and inserted into a series of pCaMGate plant binary expression vectors (Conley et al., in preparation) using the Gateway technology^®^ to generate 9 expression constructs targeting 5 different compartments (apoplast, ER, vacuole, chloroplast and cytosol) and ELP and HFBI fusions targeting the ER and vacuole compartments. To produce these vectors, first *AnpgI* was cloned into the Gateway donor vector pDONR/Zeo™ (Invitrogen, Carlsbad, USA) and the integrity of the construct was validated by sequence analysis. Using the Gateway cloning system, *AnpgI* was subsequently subcloned into the pCaMGate plant binary expression vectors (Conley et al., in preparation), derived from the pCaMterX [43] binary vector which placed the gene of interest under control of the double enhanced cauliflower mosaic virus 35S promoter [44] and the nopaline synthase (nos) terminator [45].

For constructs destined to the secretory pathway, downstream of the promoter, pCaMGate vectors harbor the tCUP translation enhancer [46], the Pr1b secretory signal peptide from tobacco [47] and the Xpress tag (for detection) followed by the *AnPgI* gene, a C-Myc detection/purification tag and a KDEL signal peptide, used for ER-retrieval. For the vacuole targeting construct, the C-terminal propeptide (CTPP) of tobacco chitinase vacuolar sorting signal [48] replaces the C-terminal KDEL; and no additonal sequence was used for secretion of *An*PGI to the apoplast. For the chloroplast-targeted construct, the transit peptide from the tobacco RuBisCo small subunit replaces the secretory signal peptide and for the cytosol no extra sequence was present downstream of the translation enhancer (Figure 1A). For the fusion constructs, ELP and HFBI are placed in-frame upstream of the c-Myc tag in the pCaMGate vectors, with a (GSSS)_3_ linker separating *An*PGI from either ELP or HFBI (Figure 1B). The final expression clones were used to transform *Agrobacterium tumefaciens* strain EHA105 [49].

### Transient expression in *N. benthamiana* and Western blot analysis

A suspension of *Agrobacterium tumefaciens* strain EHA105 carrying the expression construct was mixed with an equal amount of *Agrobacterium* culture containing the suppressor of post-transcriptional gene silencing p19 from *Cymbidium* ringspot virus [18] and co-infiltrated into leaves of 5–6 week old *N. benthamiana* plants through the stomata of abaxial leaf epidermis using a syringe [16]. Infiltrated plants were maintained in a controlled growth chamber for 2 to 9 days at 22°C, with a 16 h photoperiod. For each experiment, three leaf disks (7 mm diameter) from infiltrated tissue were collected from five different plants and ground in liquid nitrogen using 2.3 mm ceramic beads (BioSpec Products, 11079125z, Bartlesville, USA) in a TissueLyser (Qiagen^®^). Total soluble protein was extracted from the ground tissue in ice-cold phosphate- buffered saline (PBS), pH 7.4 supplemented with 0.1% Tween-20, 2% PVPP (polyvinyl polypyrrolidone), 1 mM EDTA (ethylenediaminetetraacetic acid), 100 mM sodium acorbate, 1 mM PMSF (phenylmethylsulfonyl fluoride) and 1 μg/ml leupeptin. Protein extraction from a control plant infiltrated with p19 as a negative control was also performed under similar conditions. Total soluble protein (TSP) concentration was spectrophotometrically determined using the Bradford assay [50] with bovine serum albumin as standard.

Equal volume of plant extract was separated by sodium dodecylsulphate-polyacrylamide gel electrophoresis (SDS-PAGE) (10%) and transferred to PVDF membrane. To detect the recombinant protein the membrane was incubated with primary mouse anti-C-myc monoclonal antibody (Genscript, A00864, Piscataway, USA). The primary antibody was detected with HRP-conjugated goat anti-mouse IgG antibody (Bio-Rad, 170–6516, Hercules, USA) and visualized using the ECL detection kit (GE healthcare, Mississauga, Canada) and autoradiography as described by the manufacturer. Western blots were analysed using image densitometry with TotalLab TL100 software (Nonlinear Dynamics, Durhan, USA). Intensities were determined by comparison with known amounts of a synthetic positive control protein containing a cellulose-binding domain and a C-myc tag (synthesized by Genscript, Piscataway, USA).

### Protein purification

For the protein activity assays, total soluble protein was extracted from plants producing *An*PGI 4 days post infiltration (dpi) by grinding the agroinfiltrated samples in liquid nitrogen using ceramic beads in the TissueLyser (Qiagen^®^) as previously described. Samples were mixed with six volumes (w/v) of cold PBS buffer, and the homogenate was clarified twice by centrifugation (20,000 g, 10 min at 4°C) to obtain the total soluble protein (TSP). The c-Myc purification was performed by affinity chromatography using the c-Myc tagged Protein MILD PURIFICATION KIT (MBL, 3305, Woburn, USA) according to the manufacturer’s instructions.

### Deglycosylation analysis

Enzymatic deglycosylation of transiently produced *An*PGI targeted to the apoplast, ER, and vacuole was carried out on purified protein using Endoglycosidase H (Sigma-Aldrich, A 0810, St. Louis, USA) accordingly to the manufacturer’s instructions. The Endo H specificity includes all high-mannose and hybrid type of glycans from *N*-linked glycoproteins. The digestion was carried out at 37°C for 3 h followed by SDS-PAGE under reducing conditions and western-blot analysis with anti-C-myc antibody.

### Enzyme assays

*In planta* polygalacturonase activity was assayed by estimating the amount of reducing sugar released from a 7 mm diameter leaf disk incubated in 100 μl of 50 mM of sodium acetate solution pH 5.0 at 50°C for 24 h. The amount of reducing sugar released was quantified by the DNS method [51] and the reducing sugar concentration was normalized using the assay results for control plants. Specific activity was quantitatively estimated by measuring the hydrolysis of polygalacturonic acid using an equimolar quantity of *An*PGI. The reaction was carried out in 96 well flat bottom microplates using 0.5% polygalacturonic acid substrate in 50 mM sodium acetate buffer pH 5.0 incubated at 50°C for 30 min. Galacturonic acid was used as the standard and the amount of galacturonic acid released was quantified by using the DNS method [51]. One unit of PGase activity was defined as the amount of enzyme required to release one micromole of galacturonic acid per minute per mg of protein under standard assay conditions.

### Effects of pH and temperature

The optimum enzyme pH was measured using polygalacturonic acid (0.5%) as the substrate in 50 mM acetate buffer, pH 3.0-6.0 and 50 mM sodium-phosphate buffer, pH 6.0-9.0 incubated at 40°C for 30 min. The optimum temperature was determined in the range of 20°C to 90°C in 50 mM sodium acetate buffer pH 5.0 incubated for 30 min.

### Plant cell wall saccharification

Four millimeter diameter leaf discs were collected at 4 dpi and sterilized in a 1% sodium hypochlorite solution for 5 min and washed twice with sterilized water. The plant material was incubated at 50°C for 24 hours in a filter-sterilized solution containing 50 mM sodium acetate buffer, pH 5.0. Enzymatic saccharification efficiency was determined as the amount of reducing sugars released and compared with untreated plant material using the DNS assay.

## Abbreviations

CWDE: Cell wall degrading enzyme; HG: Homogalacturonan; PG: Polygalacturonase; CBH: Cellobiohydrolase; *AnpgI*: Gene encoding polygalacturonase I protein from *Aspergillus niger*; HFBI: Hydrophobin I; ELP: Elastin-like polypeptide; TSP: Total soluble protein; SDS-PAGE: Sodium dodecylsulphate-polyacrylamide gel electrophoresis; ER: Endoplasmic reticulum; PVPP: Polyvinyl polypyrrolidone; PBS: Phosphate-buffered saline; EDTA: Ethylenediaminetetraacetic acid; PMSF: Phenylmethylsulfonyl fluoride; HRP: Horse radish peroxidase; IgG: Immunoglobulin G; DNS: Dinitro salicylic acid; tCUP: Tobacco cryptic upstream promoter; Pr1b: Pathogenesis-related protein 1b of tobacco; CTPP: C-terminal propeptide; nos: Nopaline synthase; Endo H: Endoglycosidase H; dpi: Day post infiltration.

## Competing interests

The authors declare that they have no competing interests.

## Authors’ contributions

EOP designed the research, performed the experiments and drafted the manuscript, AJC designed the pCaMGate vectors, IK provided ideas and feedback and RM conceived the study and participated in its design. EOP, IK, AJC and RM edited the manuscript. All authors read and approved the final manuscript.
